# Abiotic Stress Responsive miRNA-Target Network and Related Markers (SNP, SSR) in *Brassica juncea*

**DOI:** 10.3389/fpls.2017.01943

**Published:** 2017-11-21

**Authors:** Indra Singh, Shuchi Smita, Dwijesh C. Mishra, Sanjeev Kumar, Binay K. Singh, Anil Rai

**Affiliations:** ^1^Centre for Agricultural Bio-Informatics, ICAR-Indian Agricultural Statistics Research Institute, New Delhi, India; ^2^ICAR-Indian Institute of Agricultural Biotechnology, Ranchi, India

**Keywords:** miRNA, target genes, miR-SSR, miR-SNP, miRNA-target regulatory network, Brassica

## Abstract

Abiotic stress is one of the major factors responsible for huge yield loss in crop plants. MicroRNAs play a key role in adaptive responses of plants under abiotic stress conditions through post-transcriptional gene regulations. In present study, 95 potential miRNAs were predicted in *Brassica juncea* using comparative genomics approach. It was noted that these miRNAs, target several transcription factors (TFs), transporter family proteins, signaling related genes, and protease encoding genes. Nineteen distinct miRNA-target regulatory networks were observed with significant involvement in regulation of transcription, response to stimulus, hormone and auxin mediated signaling pathway related gene ontology (GO) term. The sucrose-starch metabolism and pentose-gluconate interconversion pathways were found significantly enriched for these target genes. Molecular markers such as Simple Sequence Repeats (SSR) and Single Nucleotide Polymorphism (SNPs) were identified on miRNAs (miR-SSRs and miR-SNPs) and their target genes in *B. juncea*. Notably, one of the miR-SNP (C/T) was found at the 5^th^ position on mature region of miR2926. This C/T transition led to the distorted and unstable hairpin structure of miR2926, consequently complete loss of target function. Hence, findings from this study will lay a foundation for marker assisted breeding for abiotic stress tolerant varieties of *B. juncea*.

## Introduction

Brassica belongs to Brassicaceae family is important sources of edible oil, vegetables, and condiments across the globe. Among various Brassica species, *Brassica juncea* is a major oilseed crop in South Asia (Woods et al., 1991). In India, *B. juncea* is grown on ~6–7 million hectares of land during the winter season under rain-fed cropping system. During various developmental stages of growth crops are exposed to several abiotic stresses such as salinity, drought, and high temperature (Zhang et al., 2014). These abiotic stresses often cause adverse effects on plant growth and development leading to substantial yield penalties (Dolferus et al., 2011).

To cope with such environmental conditions, plants have evolved with several physiological and molecular mechanisms to respond and adapt to adverse conditions. These adaptive mechanisms in plants causes altered gene expression, wherein arrays of regulatory elements are intricately involved. microRNAs (miRNAs) are key regulatory elements in various cellular responses under stress conditions (Sanan-Mishra et al., 2009; Singh and Shah, 2014). MiRNAs regulate gene expressions at post-transcriptional level by suppression of the target mRNA translation or direct cleavage (Bej and Basak, 2014). Various stress-regulated miRNAs have been identified and characterized in plants (Ramesh et al., 2015; Zhang, 2015; Ohama et al., 2016), whereas only limited reports are available in *B. juncea*. (Kanwar et al., 2012; Srivastava et al., 2013; Bhardwaj et al., 2014).

Additionally, recent evidences suggest that presence of SSRs and SNPs markers on miRNAs have potential role in the expression of quantitative traits (Ganie and Mondal, 2015). Polymorphisms as SSR or SNP on mature miRNA, may lead to disruption of interaction between putative target gene and miRNA (Ferrão et al., 2015). A line of evidence shows that SNPs are important functional sequence variations that occur at miRNA loci may have profound effects during the evolution of a species (Ehrenreich and Purugganan, 2008; Guo et al., 2008). Consequently, the expression patterns of genes could be significantly affected, sometimes leading to the appearance of transgressive phenotypes in plants (Meng et al., 2011; Liu et al., 2013; Formey et al., 2014). Therefore, identification of miRNAs and their predicted target genes together with SSRs and SNPs would be useful for designing more comprehensive studies on evaluation of the effectiveness of these markers in plant breeding.

**Figure 1:**
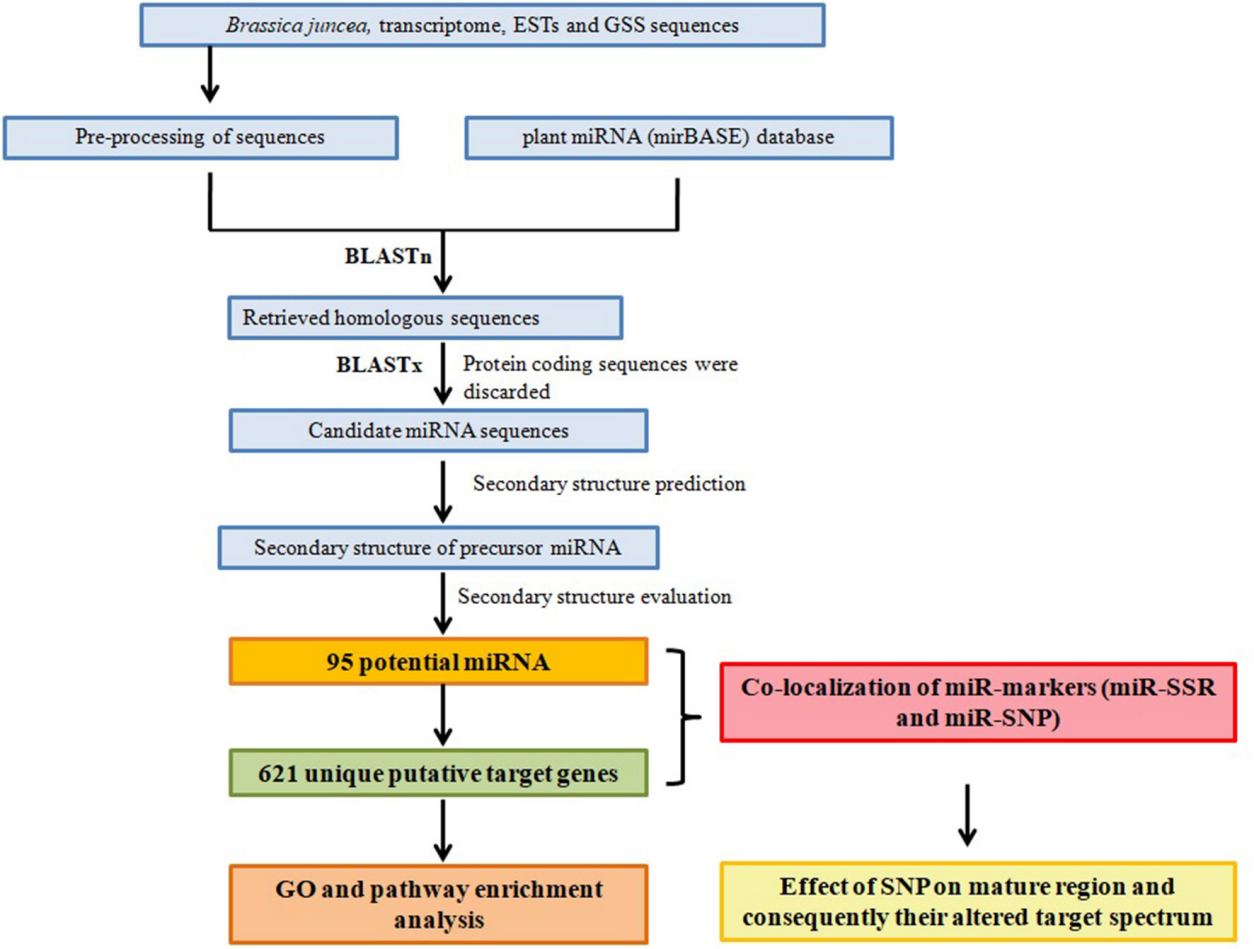
Schematic representation of the study design for miRNA, their target prediction, and miR marker identification.

Over the years, different approaches have been adapted to identify potential miRNAs in plants. The conventional approaches largely involve cloning of size-fractionated RNAs followed by Sanger sequencing and validation through experimentation (Chaudhuri and Chatterjee, 2007). However, by the virtue of highly conserved nature and similar secondary structure of the majority of miRNAs, their new homologs can be easily predicted in species of interest with the help of proper miRNA prediction algorithms and computational tools. In present study, we identified potential miRNA homologs and their target genes in *B. juncea* using available transcriptome data, ESTs and genomic sequences (GSS) following a conceptual pipeline (Figure 1). Functional classification and pathway enrichment analysis of the miRNA targeted genes was performed to understand the biological function of the predicted miRNAs. In addition, we examined the occurrence and distribution of potential SSRs and SNPs in the predicted miRNAs; miR-markers (SSRs/SNPs), and their target genes for their possible applications in marker-assisted breeding in *B. juncea*.

## Materials and methods

### Sequence dataset

Transcriptome contig sequences (GSE73201 and GSE66389), ESTs (5656), and GSS (33) of *B. juncea* were retrieved from NCBI (www.ncbi.nlm.nih.gov). These EST sequences were pre-processed by EST-trimmer (http://pgrc.ipk-gatersleben.de/misa/download/) and polyA-tails were removed. It insures that no stretches of (T)5 or (A)5 were present on 5′ or 3′ ends of trimmed sequences in a range of 50 bp. To exclude low quality sequences, ESTs with less than 100 bp and more than 800 bp were discarded and clipped at 3′ end (Thiel et al., 2003). The processed and filtered sequences were then assembled using CAP3 (Huang and Madan, 1999) program. Transcriptome data encompassing 2,148,658 and 933,376 transcript sequences respectively from GSE73201and GSE66389, 799 assembled ESTs, 2774 ESTs singleton from EST and GSS were further subjected in miRNA prediction pipeline.

### Identification of potential miRNA in *B. juncea*

All available plant miRNAs and their precursor sequences were retrieved from miRBase v 20.0 repository; a database of published miRNA (Griffiths-Jones, 2004). Non-redundant set of miRNA sequences were used for BLASTn (mismatch < 1, with no gap and E-Value 0.01) homology search against total transcriptome contigs, assembled EST contigs, singleton and GSS sequences. Further BLASTx was performed against non-redundant (NR) protein database to remove protein-coding sequences (Altschul et al., 1997).

### miRNA secondary structure prediction

The secondary structures of non-redundant miRNA sequences were generated using Zuker folding algorithm implemented in Mfold (http://mfold.rna.albany.edu/?q$=$mfold/download-mfold/) with default parameters (Zuker, 2003). Predicted miRNA secondary structure should have higher Minimal Free Energy Index (MFEI) folding and negative Minimal Folding Free Energy (MFE) to differentiate it from other small RNAs. Sequences satisfying Meyers rules were considered as potential miRNA (Meyers et al., 2008). The predicted mature miRNAs had no more than one nucleotide substitutions compared with a known reference mature miRNAs, (i) it must fold into proper stem-loop hairpin structures, (ii) it should be present on one of the arm of hairpin, (iii) no more than four mismatch in predicted miRNA and the miRNA^*^ (on opposite arm of mature region), (iv) it must not contain more than one bulges, (v) the size of bulges are not more than two, (vi) the predicted secondary structure must contain high negative MFE and high MFEI values.

### Prediction of potential target genes of miRNAs

In order to understand the biological functions of miRNAs, putative target genes were identified by using psRNA Target server (http://plantgrn.noble.org/psRNATarget/) against available *B. juncea* transcript sequences and *B. rapa* unigenes, which is close to *juncea* (as it contains AA genome) (Mun et al., 2009). Functional annotations of target genes were retrieved through BRAD Database (http://brassicadb.org/brad/) (Cheng et al., 2011). Based on UPE (unpaired energy) energy and inhibition process, miRNA-target network were built among miRNA, and their target genes using cytoscape software (Shannon et al., 2003). Gene Ontology enrichment analysis was performed using agriGO (Du et al., 2010) by Singular Enrichment Analysis (SEA) with hypergeometric statistical test method and Bonferroni multi-test adjustment method (significance level < 0.05).

### Simple sequence repeats (SSR) mining

Simple sequence repeats (SSR) mining was performed using MISA (microsatellite search module) with 2–6 bp repeats as described by Thiel et al. (2003).

### Identification of single nucleotide polymorphism (SNP)

Putative SNP/indels were extracted by using AutoSNP pipeline for SNP discovery (Barker et al., 2003). The programme first makes a cluster of sequences followed by contigs formation of highly homologous sequences. Each of the contig was analyzed for presence of SNP sites. For identification of miR-SNP, precursor miRNA sequences were subjected for SNPs detection with low E-value and occurrence in more than five sequences. SNPs on target genes were identified by comparing it with reported SNPs from *B. juncea (AABB)* (Paritosh et al., 2014) and *B. napus (AACC)* (Trick et al., 2009). Further, we utilized Protein variation effect analyser (PROVEAN) (Choi and Chan, 2015) and PANTHER (Thomas et al., 2003) tool to predict the change in the biological functions of a target protein due to an amino acid substitution or indel based on the sequence clustering and alignment-based scoring. In PROVEAN, variants with scores less than −2.5 were considered deleterious and greater than −2.5 as a neutral variant (Choi and Chan, 2015).

## Results

### Identification of potential miRNAs in *B. juncea*

Homology search against miRBase as well as a series of manual inspection, evaluation, and filtration processes was performed. Total of 95 unique potential miRNAs (miR1114, miR156h, miR2926, miR3434-3p, miR408, miR5015, miR5021, miR536f, miR5658, miR854a-e), sequences belonging to 32 miR-families were identified in *B. juncea* (Table S1A). All miRNA sequences were 19–24 nt long, with Watson-Crick or G/U base pairing and stable minimal folding free energy (MFE). Precursor sequences of predicted miRNA from ESTs were mapped to *B. juncea* genome and transcriptome to validate they are real miRNAs (Table S1B).

### Target genes prediction for identified miRNAs

Target gene prediction is a key step for determining biological function of miRNAs. A total of 906 unique potential target genes were predicted for identified miRNAs using *B. juncea* transcript sequences and *B. rapa* unigenes (Tables S2A–C). Many of the identified potential miRNA targets were member of several transcription factor (TFs) family, signaling related genes and growth regulating factors. Especially, miR156, miR171 miR5021, miR2926, miR393, miR164, miR408, and miR854 displayed a striking inclination to TFs target genes (Table S2).

Widely studied miR156 predicted to target many squamosa promoter binding protein-like (SBP domain) TF, ATP binding cassette subfamily, F-box family protein, F-box/RNI-like/FBD-like domains-containing proteins. MiR160 family was predicted to target auxin response factors (10, 16, and 17). Similarly miR393 targeted auxin signaling F-box (2 and 3) and targets of miR5021 were WRKY, CPK2 (calmodulin-domain protein kinase), ARR2 (Arabidopsis response regulator 2), MYB101, heat shock protein binding, TIR1, and NAC. MiR408 targeted 22 genes, including chloroplast heat shock protein 70-1, ASPARTATE KINASE 3, F-box family protein, and SWAP (Suppressor-of-White-Apricot) (Table S2).

MiR2926 targeted ATP binding, kinase, ATGWD3, elongation factor 1-beta/EF-1-beta, APUM5 (Arabidopsis Pumilio 5), peroxisomal membrane protein (PMP36), DNAJ heat shock family protein, zinc finger, and sterile alpha motif (SAM). The miR5015 received four target genes such as, SPX (SYG1/Pho81/XPR1), zinc finger (C3HC4-type RING finger) protein-related, PGIP1 (Polygalacturonase Inhibiting Protein 1), and PHD finger family protein, PHYC (phytochrome defective c) protein histidine kinase. All these miRNA-target annotation showed their involvement in various biological processes such as TF mediated and/or signaling related pathways.

### Functional and pathway enrichment analysis of miRNA target genes

**Figure 2:**
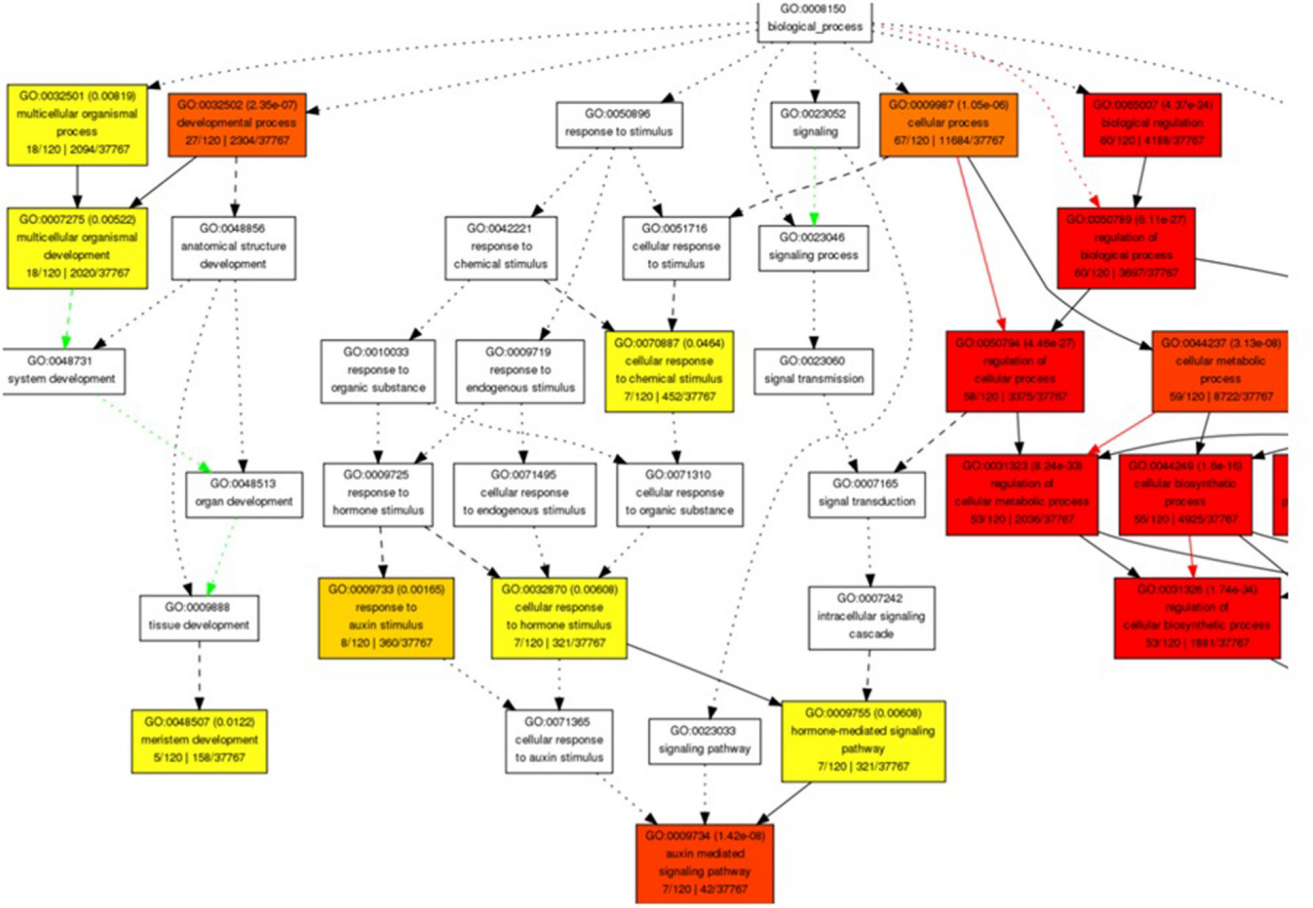
Snapshot of significantly enriched biological process Gene Ontology terms for predicted target genes of miRNA in *B. juncea*.

The miRNA-target genes ontology (GO) enrichment showed their association to signal transduction and several cellular and metabolic processes. We observed the regulation of transcription, developmental process, nitrogen compound metabolic process, macromolecule biosynthetic, hormone stimulus, and auxin mediated signaling pathway related biological processes GO term with significant enrichment (Figure 2). Moreover, transcription regulator activity binding and DNA binding were noted to be significantly enriched molecular function GO categories. Enrichment of nucleus, intracellular membrane-bounded organelle, integral to membrane, and intrinsic to membrane were observed with significance cellular component GO terms (Figures S1A–C).

Pathway analysis of miRNA target genes showed significantly enriched pathways under environmental information processing such as signal transduction, carbohydrate metabolism, starch and sucrose metabolism and pentose and glucuronate inter-conversions pathways (Figures S2A,B). Exploring the results further showed, target of miR396, miR5658, miR854e, miR854a miR408, miR5021 were involved in starch and sugar metabolism. While miR408 and miR5021 regulated targets genes annotated as glycosyl hydrolase family protein were also involved in pentose and gluconate inter-conversion pathway. SPL target predicted for miR156 were found involved in plant hormone signal transduction pathways.

### Co-localization of SSR markers on pre-miRNA (miR-SSRs) and their target genes in *B. juncea*

SSR motifs on 95 pre-miRNA sequences were shown three type of SSR motifs were co-localized on 10 pre-miRNAs (Table S3A). These SSRs on 10 pre-miRNAs were from five miR families such as miR824, miR396, miR5021, miR156, and miR5015. Total of six SSR motifs were overlapped on mature miRNA region. Most of the SSR motifs were trinucleotide with frequent CTT and one of the C^*^ from miR156h (Figure S3A).

Mining of SSR motifs on target genes showed 700 SSR motifs in 621 target genes with maximum frequency of trinucleotide repeats (Tables S3B,C). Results depicted that 4 mononucleotide repeats, 5 dinucleotide repeats, 656 trinucleotide repeats, 12 tetranucleotide repeats, 1 pentanucleotide repeats, and 22 nucleotide repeats Figure S3B were present on targets. It is observed that 17 of the miRNA target sequences have more than one SSR motifs. Total of 14 compound SSR (C^*^ and C) motifs were identified from miRNA target genes.

### Occurrence of SNPs on pre-miRNA (miR-SNPs) and their effect

**Figure 3:**
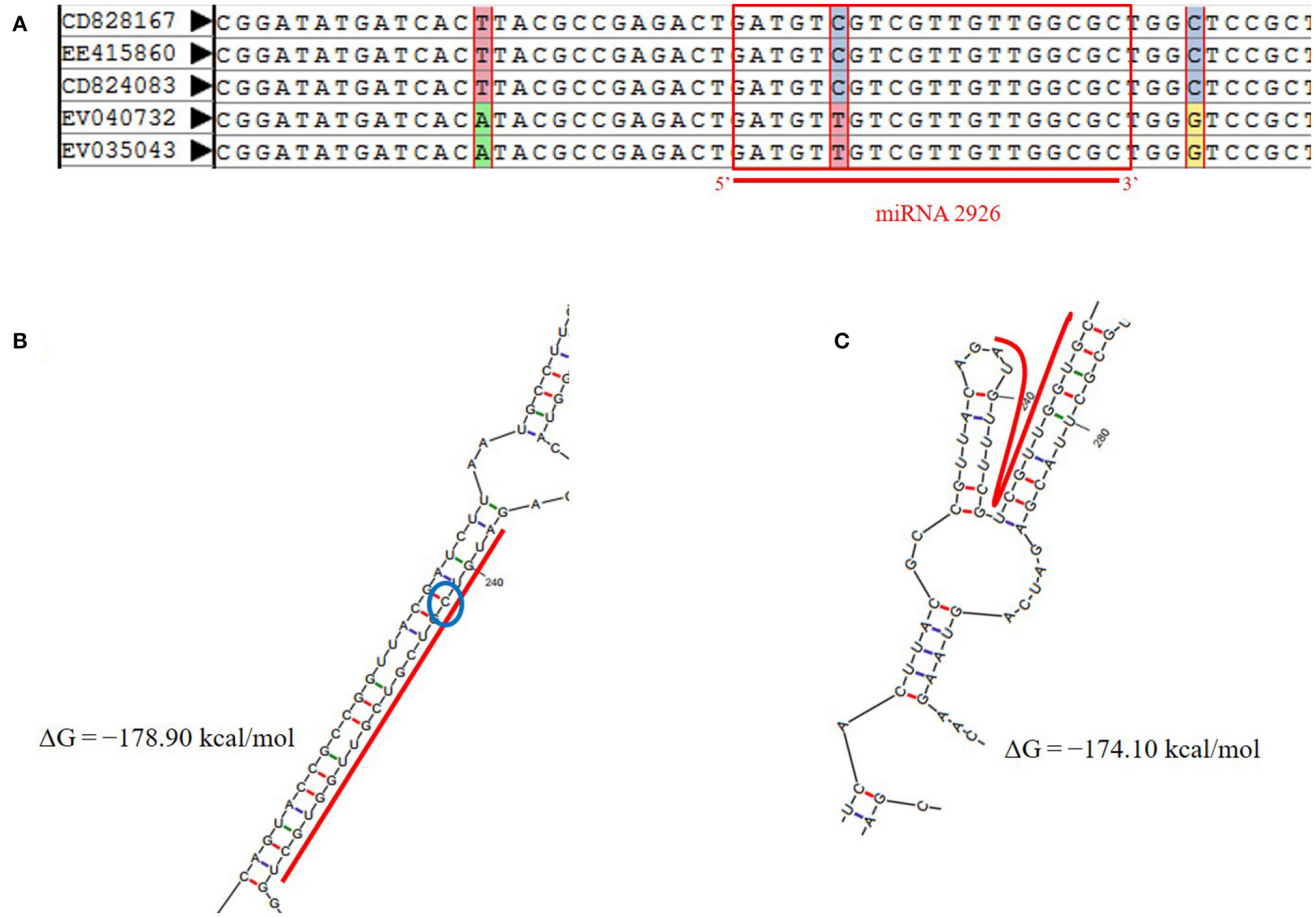
**(A)** miR-SNP (C-to-T transition at 5th position) on mature region of miRNA-2926 in *B. juncea*. **(B)** Secondary structure of miRNA-2926 without SNP, ΔG = −178.90 kcal/mol. **(C)** Secondary structure of miRNA-2926 with miR-SNP (C/T) (ΔG = −174.10 kcal/mol). The blue circle shows SNP position.

Reliable SNPs were retrieved on 95 pre-miRNAs and mature miRNA using integrated AutoSNP pipeline with ESTs mapped from more than one genotype (Barker et al., 2003). Total 34 SNPs were found, with greatest number of transition i.e., 14 (46.47%) (Table S4A). Indels were localized on three precursor sequences of miR854, miR824, and miR171 (Table S4A). The SNPs identified in miR854 were located on flanking to the downstream of mature region. However, SNPs on miR2926 were located on the precursor as well as mature region. MiR-SNP (snpID: rs54273) with transition (C/T) type at 5th from 5′ end of mature region of pre-miR2926 were noted (Figure 3A).

Further, effect of miR-SNP was evaluated on modified structure of miR2926 due to base transition (C to T) at 5th position (Figure 3B). Evaluating the possible impact of SNP on pre-miRNA secondary structure, ΔG, and MFEI were also calculated. Loss of stability has been observed with increased ΔG from −178.90 to −174.10 as well MFEI from −0.5 to −0.476 of the secondary structure with this base transition from C to T. Figure 3C clearly shows the structural variation due to the transition i.e., loss of miRNA-miRNA^*^ duplex. This exercise demonstrated that presence of SNP on miR2926 destabilized pre-miRNA structure. This consequently affects their target genes. Hence, with the aim to better understand the alteration in target spectrum due to SNP, putative targets of miRNA with transitioned nucleotide (C to T) were searched using psRNA Target tool. Notably, no target genes were found for miR2926 with miR-SNP; reflecting as complete loss of function of target genes. These results justifying our *in-silico* finding of distorted secondary structure.

### SNPs on target genes of miRNAs and their deleterious effect

**Figure 4:**
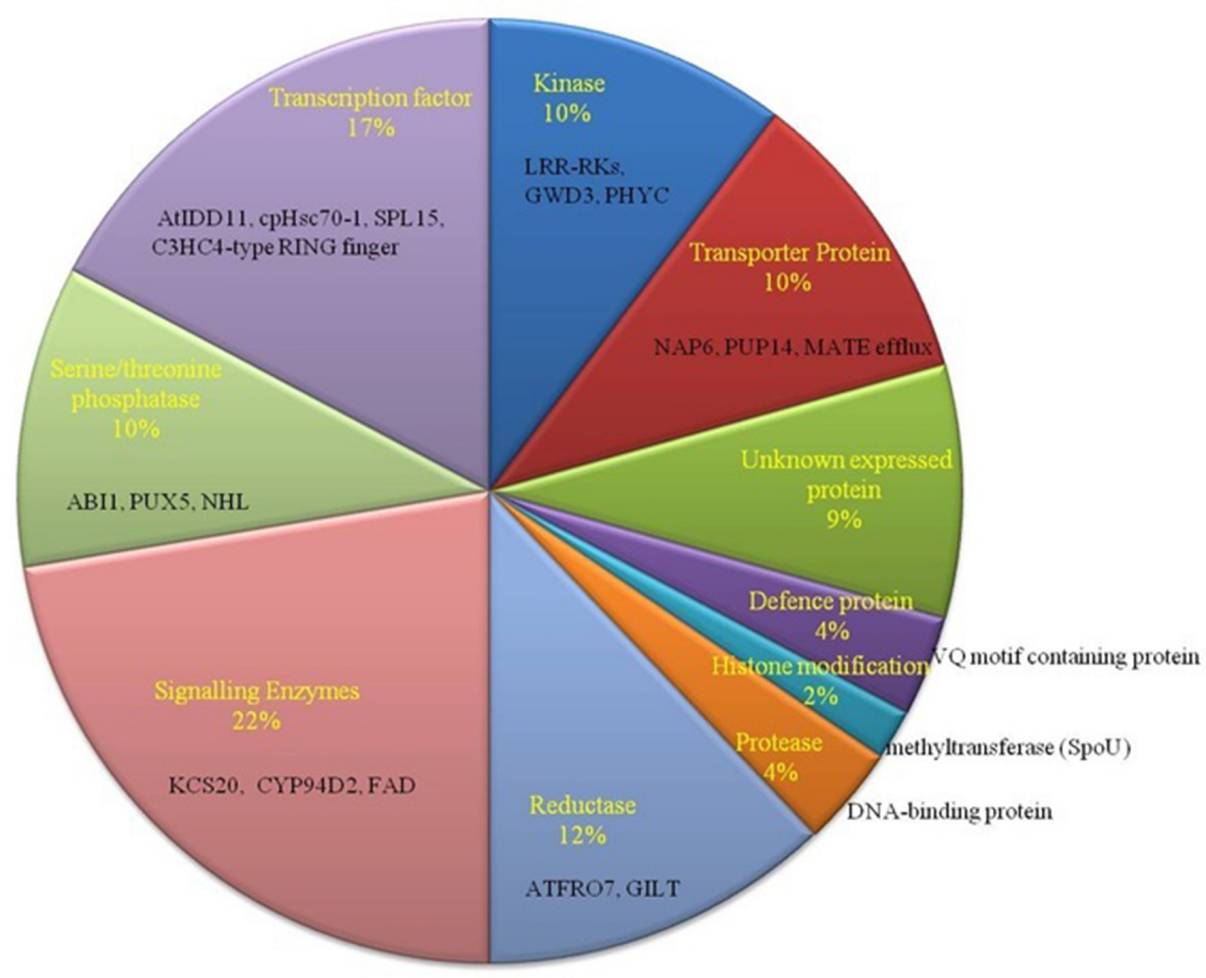
Functional classification of miRNA-target genes with identified SNPs. Most of the genes belong to signaling enzymes such as KCS20, CYP94D2, and FAD (22%).

To identify SNPs on target genes of our predicted miRNAs in *B. juncea (AABB)*, they were mapped to the already observed genes with SNPs in *B. juncea (AABB)* (Paritosh et al., 2014) and *B. napus (AACC)* (Trick et al., 2009). Total of 100 targets (79 matched Ids with *B. napus* and 21 with *B. juncea)* were found having SNPs, where C-to-T substitution with a greatest frequency (Tables S4B,C). These targets with SNPs mapped have been found to be regulated by miR854 (mostly), miR171, miR408, miR156, miR2926, miR5658, miR5015, miR3434, and miR5021. The functional annotation of 100 target genes with SNPs revealed association to signal-enzymes, followed by TF family genes that known to play important role in abiotic stress condition (Figure 4). Further, deleterious consequences of SNPs found on target genes from *B. juncea* were investigated and found 13 synonymous and 5 non-synonymous SNPs. Notably, two SNPs on Bra039026 (V208G) and Bra020200 (S330Y) were predicted having deleterious effect with PROVAEN score −6.6 and −3.3, respectively and also “probably damaging” and “probably benign” respectively from PANTHER.

### miRNA-mediated regulatory networks

**Figure 5:**
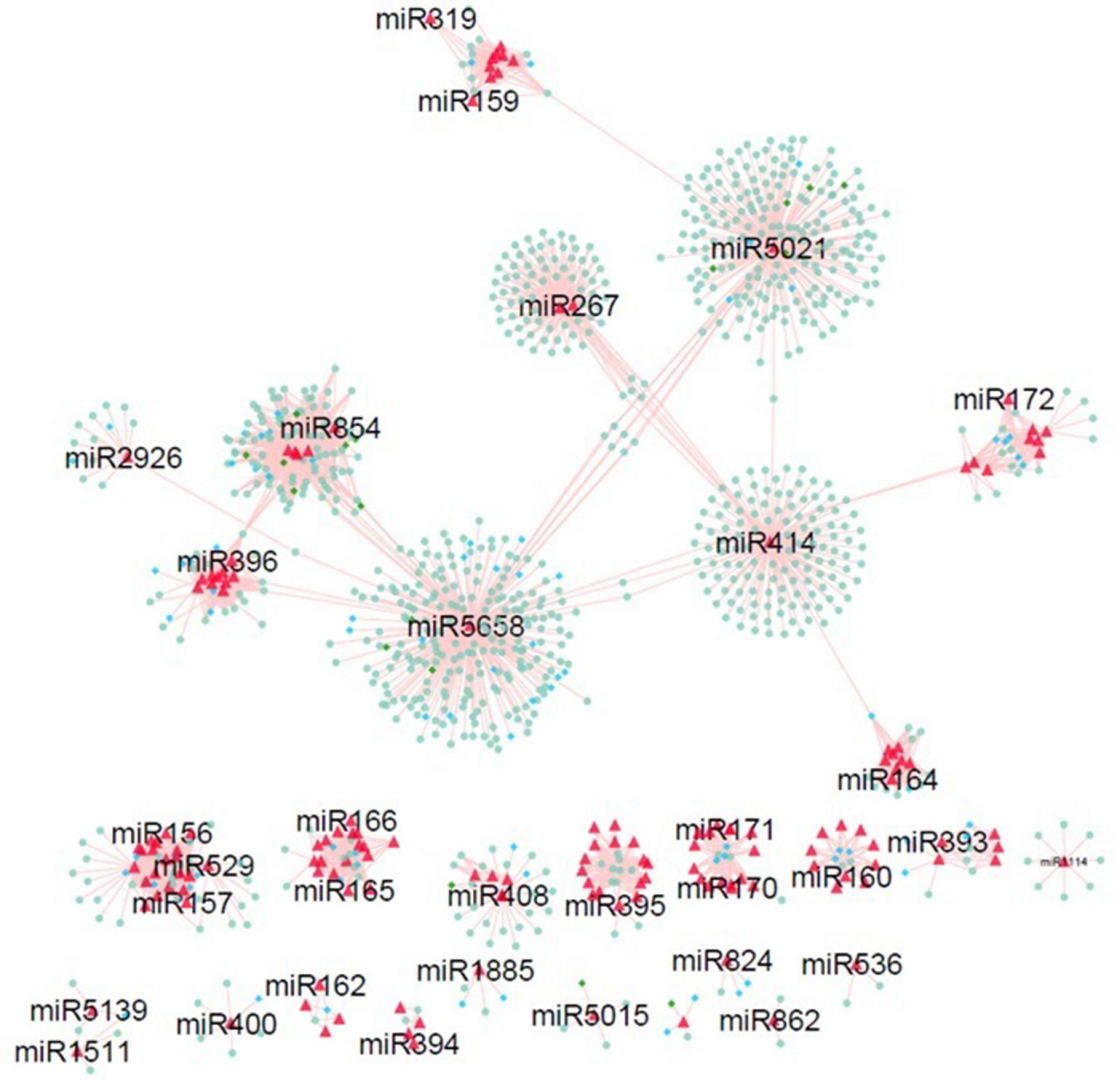
MiRNA-target genes regulatory network identified in *B. juncea*. Red triangle nodes in network represents miRNAs. Nodes in diamond shape denote target genes having SNP mapped from *B. juncea* (green nodes) and *B. napus* (blue nodes).

In order to explore the relationship among the miRNAs and their targets, miRNA-mediated regulatory networks were constructed. Nineteen independent networks showing multiplicity behavior of miRNA i.e., one miRNA can target more than one genes (Figure 5). Exploring these results revealed miR156 sharing target genes with miR157 and miR529. MiR854 regulatory network connected with miR5658 network with common target genes (Bra006283, Bra011043, Bra030266, Bra034179, Bra005011, and Bra001582) involved in transmembrane transport and zinc ion binding. Common target genes (Bra027592, Bra035905, Bra040355, Bra020820, Bra001710, and Bra014818) with functions as aspartic-type endopeptidase activity, lipid glycosylation, and proteolysis for miR5658 and miR5021. It indicates the co-operative behavior of these target genes, where one gene can be controlled by more than one miRNA (John et al., 2004). Many other miR-networks were found to be independent to each other, where one miRNA regulate multiple targets showing their multiplicity behavior. Further, mapping of the target genes reported bearing SNPs on the network was done. Interestingly, network showed maximum of SNPs on miR854 regulated target genes (green circle nodes on network in Figure 5), followed by miR5021 and miR5658 reported in *B. juncea* (Paritosh et al., 2014).

## Discussion

Computational prediction of miRNAs using transcriptome, EST, and GSS sequences is rather a prompt and cost effective approach to identify potential miRNAs (Frazier et al., 2010; Kim et al., 2011; Katiyar et al., 2012; Srivastava et al., 2013; Bhardwaj et al., 2015). In current study, 95 potential mature miRNAs in *B. juncea*, utilizing comparative genomics-based approach were predicted. A notable aspect of the present study was addition of few new *B. juncea* miRNAs sequences variants for miR396, miR408, miR160, and miR166 as compared to the results mentioned in previous studies (Yang et al., 2013; Bhardwaj et al., 2014) (Table S5).

The complementarities between the miRNA and their target genes provided the hierarchy of biological process regulation. The predicted miRNA targets genes coding of proteins such as, TFs, transporters, defense, and stress responsive signaling pathways. The expression of important TF families like MYB, BHLH, and zinc finger in relation to miRNAs has been reported to be involved in hormonal homeostasis (Sorin et al., 2005; Siemens et al., 2006). We compared the expression of target genes using *Arabidopsis* homologs with transcriptome data available for *B. juncea* (Table S6) (Sharma et al., 2015; Sinha et al., 2015; Srivastava et al., 2015). We observed genes encode nuclear response regulator that acts as a negative regulator in cytokinin-mediated signal transduction were down regulated under salinity stress (Table S6A). Whereas, number of target genes were observed down regulated under arsenic stress (Table S6B). This may indicate the upregulation of upstream miRNAs under these stress condition.

### Predicted *B. juncea* miRNA-target regulatory network might involve in abiotic stress response and signaling pathways

The target genes of miR156 such as CAT, ABCB, CBS, and SPL showed their involvement in stress response related biological processes. SPL has been studied in response to stress and temporal expression changes during leaf development in *B. juncea* and other plants (Wang et al., 2009; Xie et al., 2012; Stief et al., 2014). Other target genes such as ABCB, CAT5, and CBS reported to regulate signaling pathways under stress conditions (Kushwaha et al., 2009). Role of miR156h has been reported to be involved in hormonal homeostasis, abiotic stress responses, tissue development and cytoplasmic male-sterility in *B. juncea* as well as other plants (Achard et al., 2004; Sunkar and Zhu, 2004). It is reported that miR156 also expressed during club root formation in canola (Verma et al., 2014) and turnip mosaic virus infection in *Arabidopsis* (Kasschau et al., 2003; Navarro et al., 2008) Overexpression of miR156b and miR156h resulted in dwarfism as well as reduced panicle size in rice (Chen et al., 2015). These findings are obvious true as phytohormones functions to coordinate plant growth and development as well as for mitigating the effect of stress as studied in *B. juncea* and several others (Hasanuzzaman et al., 2013; Wilson et al., 2014).

MiR854 target networks are mostly involved in signaling pathways, which is in consistent with previous study. Srivastava et al. reported high expression of miR854 in response to arsenic stress in roots (Srivastava et al., 2013) and low expression under jasmonic acid supplementation (Srivastava et al., 2013) in *B. juncea*. They described that under arsenic stress, the altered expression of miR854, led to the interplay of JA, IAA, and miRNAs, where suppressing its expression lead to the increased expression of its target genes in *B. juncea*. Notably, LSD1 was reported to be target gene of miR854, functions as marker for JA signaling, which acts as antagonistic transcriptional regulators to control attenuation of cell death through regulation of ROS (reactive oxygen species) (Tamaoki et al., 2008).

Most of the targets of miR5021 such as WRKY, MYB, and ARR2 have regulatory function under stress condition. Previous studies have shown MYB protein was targeted by miR5021 in *Catharanthus roseus* (Pani and Mahapatra, 2013) and it has proven role in abiotic and boron stress response (Katiyar et al., 2012; Ozhuner et al., 2013; Smita et al., 2015). Further, miR1114 has been studied for their role during seed development in *B. napus* (Huang et al., 2013) and their target genes such as kinase and F-box have studied to play role in seed development in *B. juncea* and *B. napus* (Fei et al., 2007; Mandal et al., 2011). The target genes of miR5015 such as leucine zipper, HSF, PGIP1 etc. were also observed in carrot, which are having significant role in response to abiotic stress and in defense response (Berger et al., 2000; Barozai et al., 2013).

The pathway enrichment analysis of target genes showed starch—sucrose metabolism and pentose-glucuronate inter-conversions pathways with highest significance.

**Figure 6:**
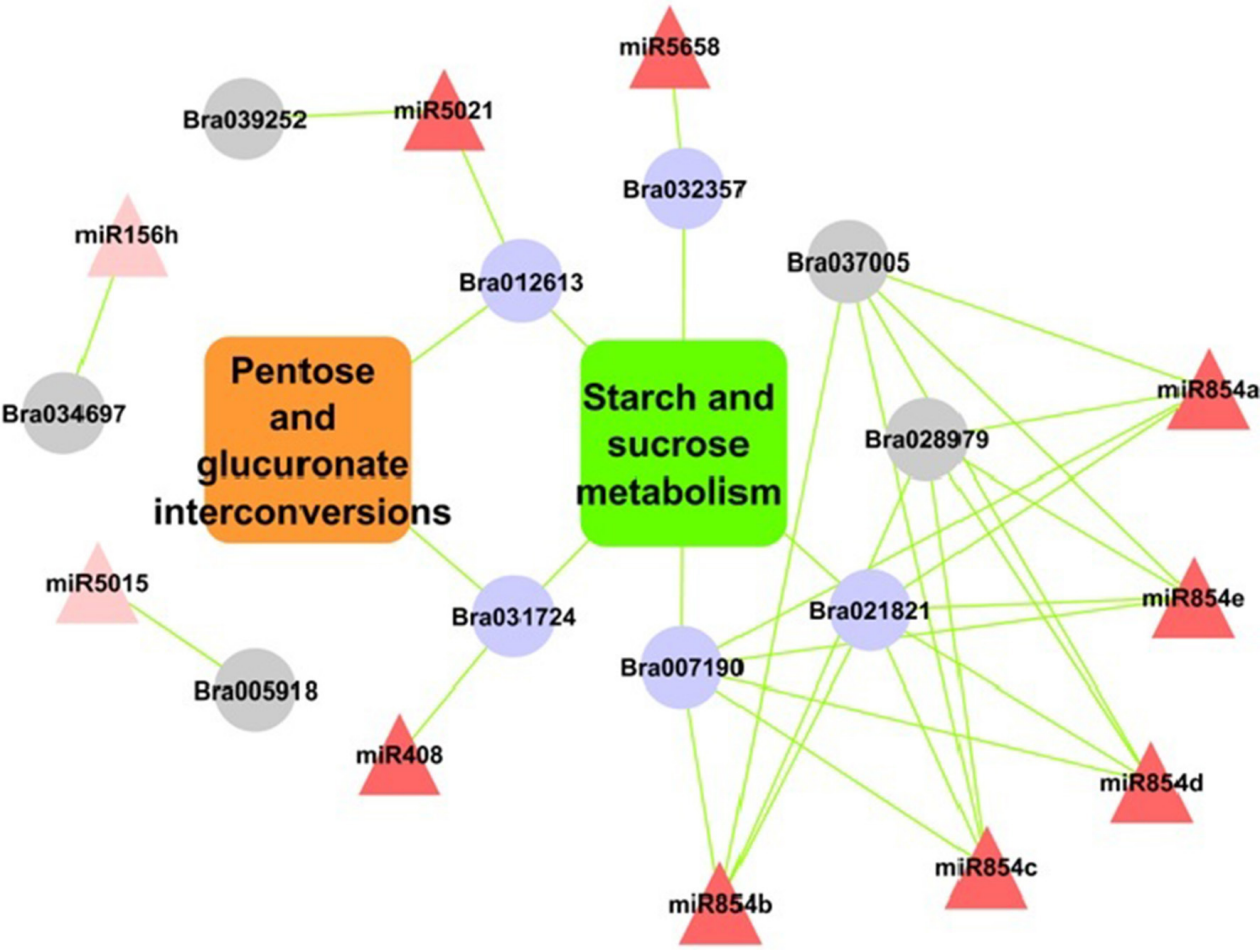
Relationship of miRNAs and target genes in starch-sucrose pathway and pentose and glucuronate interconversions in *B. juncea*. Nodes in red colors are miRNAs; rectangular boxes are pathways and in purple are significantly enriched target genes. Nodes in gray color are with “glycosyl related terms.”

These targets were majorly annotated as glycosyl hydrolase family protein and targeted by five of the miRNA (miR5658, miR854e, miR854a, miR408, and miR5021) (Figure 6). These pathways are part of carbohydrate metabolism, which is an important biochemical processes in plant growth and stress tolerance (Zhou et al., 2010). Previous study showed that the coding protein β-fructofuranosidase target genes down-regulated by miR408, takes part in starch and sucrose metabolism in rice (Zhou et al., 2010). Additionally, miR3434 have been reported for its role in starch metabolism in *Arabidopsis* leaf (Ingkasuwan et al., 2012). Consistent to our findings discussed above it may be noted that these miRNAs targeted genes and their regulatory cascade showing their significant involvement in starch and sucrose metabolism pathway during abiotic stress response.

### Heat stress related miRNA-target regulatory network in *B. juncea*

Heat stress at seedling stage is crucial in India, whereas, it is a major problem during flowering stage in major mustard growing countries such as China, Australia, Canada (Angadi et al., 2000; Sharma and Sardana, 2013). Therefore, heat stress in *B. juncea* has been a major problem especially in Asian countries.

**Figure 7:**
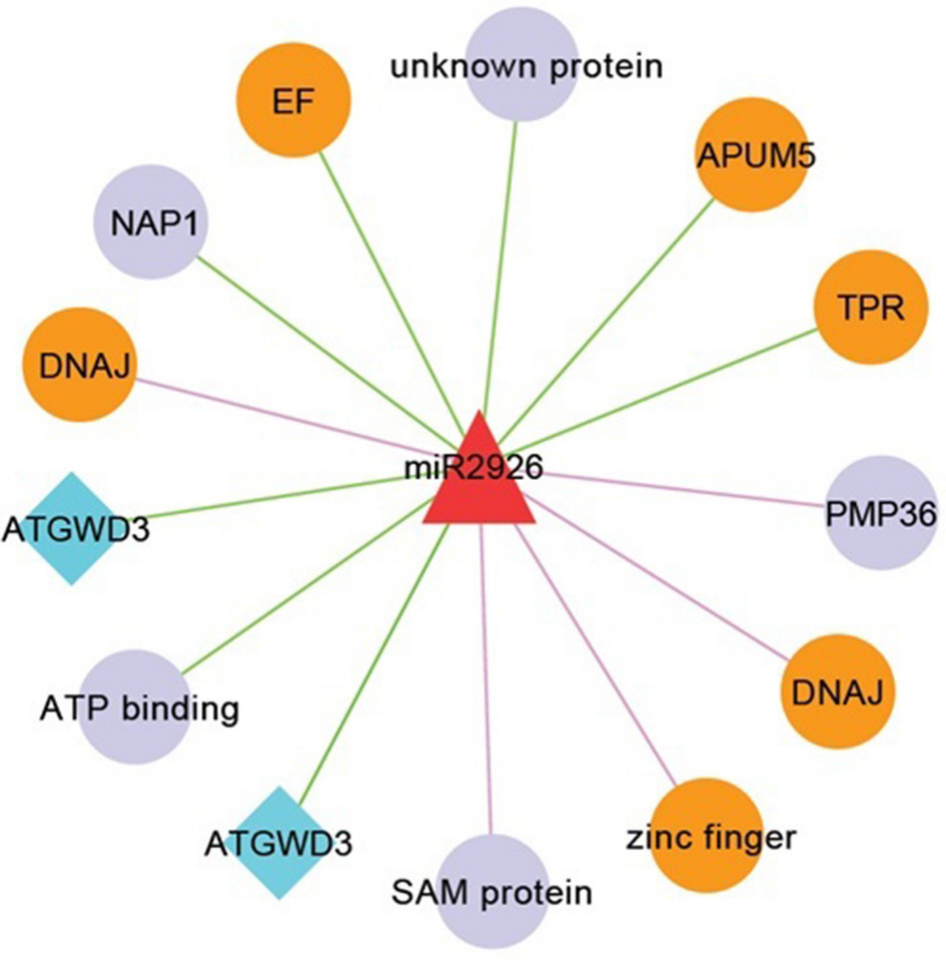
Network consists of miR2926 and their target genes. Nodes in orange color are known to be involved in heat stress. Nodes in diamond with blue color denotes that target genes having SNP mapped from *B. napus*. Edges in pink and green color, respectively showing translation and cleavage type of inhibition.

MiR408 and their target genes such as heat shock protein 70-1 and ASPARTATE KINASE etc. were well studied in response to heat stress in checkpea and rice (Mutum et al., 2013; Hajyzadeh et al., 2015). These results are also consistent with previous study in wheat and *Populus euphratica*, where, it was observed that miR408 have differential expression in response to heat stress also (Ozhuner et al., 2013). These results indicated that heat stress drive change in expression of many TFs and kinases which serve as key components of signal transduction pathways (Bhardwaj et al., 2015). Also, miR408 were identified and checked for their response in zinc deficiency in *B. juncea* roots (Shi et al., 2013) playing important role in heat stress. Most of the targets of miR2926 such as APUM5, DNAJ, zinc finger, TPR, and elongation factor were reported to be involved in response to heat stress (Bekh-Ochir et al., 2013; Bogamuwa and Jang, 2014; Huh and Paek, 2014) (Figure 7). Interestingly, miR2926 has been studied in response to heat stress in *Saccharina japonica* (Liu et al., 2015). In general, on the basis of earlier functional analysis we hypothesized, that miRNA target networks consist of miR408 and miR2926 were predominantly related to heat stress responses.

### MiR-markers in *B. juncea*

It has been reported that markers present in UTRs and introns can modulate gene expression (Li et al., 2002). Therefore, in the present study co-localization of SSR and SNP markers were discovered on the precursor as well mature region of identified miRNAs (miR-SSR and miR-SNPs) in *B. juncea*. We observed, miR5021, miR5015, and miR156h were found with trinucleotide SSR repeats in highest frequency. Therefore, these miRNAs with traits (abiotic stress) specific SSRs may be utilized further in marker assisted breeding program in *B. juncea*. In a recent study, trait-specific SSRs were identified showing salt response in rice (Ganie and Mondal, 2015). Although, the development of EST-SSR markers was reported in plant species and *Brassica* genotype for marker assisted breeding (Hopkins et al., 2007; Gupta et al., 2014). But limited information is available till date about the miR-SSR in *B. juncea*, despite it is being a major oilseed crop (Ganie and Mondal, 2015). A preliminary analysis to verify the presence of SSR motifs in primary-miRNAs of black pepper was reported (Joy and Soniya, 2012). A very good attempt was taken by a group for analyzing SSR motifs in 8619 premature-miRNAs from 87 species, mostly were from 3 to 4 bp repeats and suggested SSRs are an important component of pre-miRNAs (Chen et al., 2010). These results can be further utilized for marker assisted breeding programme, as it directly or indirectly affect the expression of miRNA and subsequently its target expression (Li et al., 2002).

Additionally, functional SNPs can cause apparent phenotype changes or trait variations (Shastry, 2009), which mainly dispersed in sequence regions with less conservation, such as intronic and intergenic regions, but fewer in functional regions, such as coding sequences (CDSs) and regulatory elements (Castle, 2011). In current study, high frequency of C/T transition noted in consistent with previous study (Low et al., 2014; Rawlings-Goss et al., 2014) (Table S4A). It has been also reported that, C/T transition at the target site enhances the repression of TGFBR2 by hsa-miR-34b^*^ (Ahluwalia et al., 2012). We identified two SNPs with deleterious effect on genes encoding methyltransferase and phosphoglycerate mutase targeted by miR3434 and miR5658. Methyltransferases such as HEN1 has been studied to involve in plant miRNA biogenesis (Tkaczuk et al., 2006; Baranauske et al., 2015). Phosphoglycerate mutase studied to have critical roles in vegetative growth in *Arabidopsis thaliana* (Zhao and Assmann, 2011). One SNP on mature region of miR2926 that de-stabilizes the secondary structure and consequently the complete loss of function (Figure 3). Our results showed miR2926 role in heat stress, in consistent with Liu et al. reported the role of miR2926 in response to heat stress in *S. Japonica* (Liu et al., 2015). Therefore, identified SNP marker on mature miR2926 region is a researchable area and seeks further validation to achieve trait specific goal. These SNPs can be utilized further for fine mapping of important agronomic traits and will shed light on the diversification of *Brassica* species.

## Conclusion

Identified miRNAs and their target genes are potentially involved in abiotic stress response, developmental stages and sugar-starch metabolic pathway in *B. juncea*. The functional regulatory networks of miRNAs provided insight into the details of particular miRNA functional comprehensions. Conclusively, this modular dissection and analysis unravel several functional and their molecular decoding. Co-localization of miR markers (miR-SSR and miR-SNP) on mature miRNA and their target genes associated to abiotic stress in *B. juncea* revealed their utility for trait specific marker assisted breeding program. Occurrence of SNP at mature miR2926 had changed the stability of miRNAs secondary structures as well as the miRNA-target interactions. Thereby affecting the miRNAs biogenesis and causing the complete loss of function. Overall, the finding from present study will contribute to further deepen understanding of the effect of markers on the evolution of miRNAs in *B. juncea*. This will lead to abiotic stress varietal development through marker assisted breeding and mitigation of faster changing climatic conditions.

## Author contributions

IS and SS initiated the research, performed the downstream analyses, interpreted the results and drafted the manuscript. IS, SS, SK, DM and AR interpreted the results. SK, BS, and DM conceived the idea of the study and drafting the manuscript. All authors have read and approved the manuscript for publication.

### Conflict of interest statement

The authors declare that the research was conducted in the absence of any commercial or financial relationships that could be construed as a potential conflict of interest.

## Supplementary material

Figure S1 Gene ontology **(A)** biological process, **(B)** cellular component, **(C)** molecular function enrichment analysis of target genes for predicted miRNA in *B. juncea*. Darker the color more significant is the term.

Figure S2 Enriched pathway **(A)** starch and sucrose metabolism, **(B)** pentose and glucuronate interconversions for target genes for predicted miRNA in *B. juncea*.

Figure S3 Distribution of simple sequence Repeats (SSRs) on **(A)** miRNA precursor sequences and **(B)** target genes in *B. juncea*.
