# Supplementary figures and images for: Abiotic Stress Responsive miRNA-Target Network and Related Markers (SNP, SSR) in Brassica juncea
Source: Front Plant Sci. 2017 Nov 21;8:1943. doi: 10.3389/fpls.2017.01943 (PMC5702422; doi:10.3389/fpls.2017.01943)

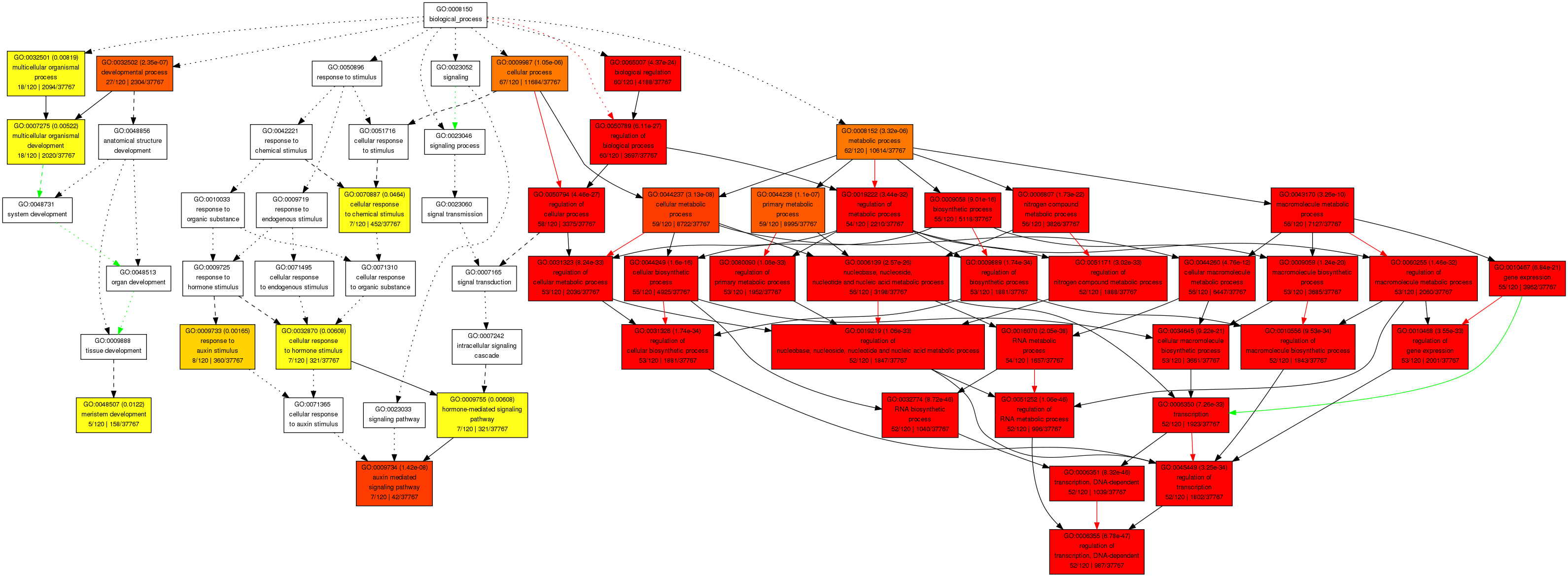

Supplement: Figure S1 — Gene ontology (A) biological process, (B) cellular component, (C) molecular function enrichment analysis of target genes for predicted miRNA in B. juncea. Darker the color more significant is the term. [file FigureS1a.PNG]

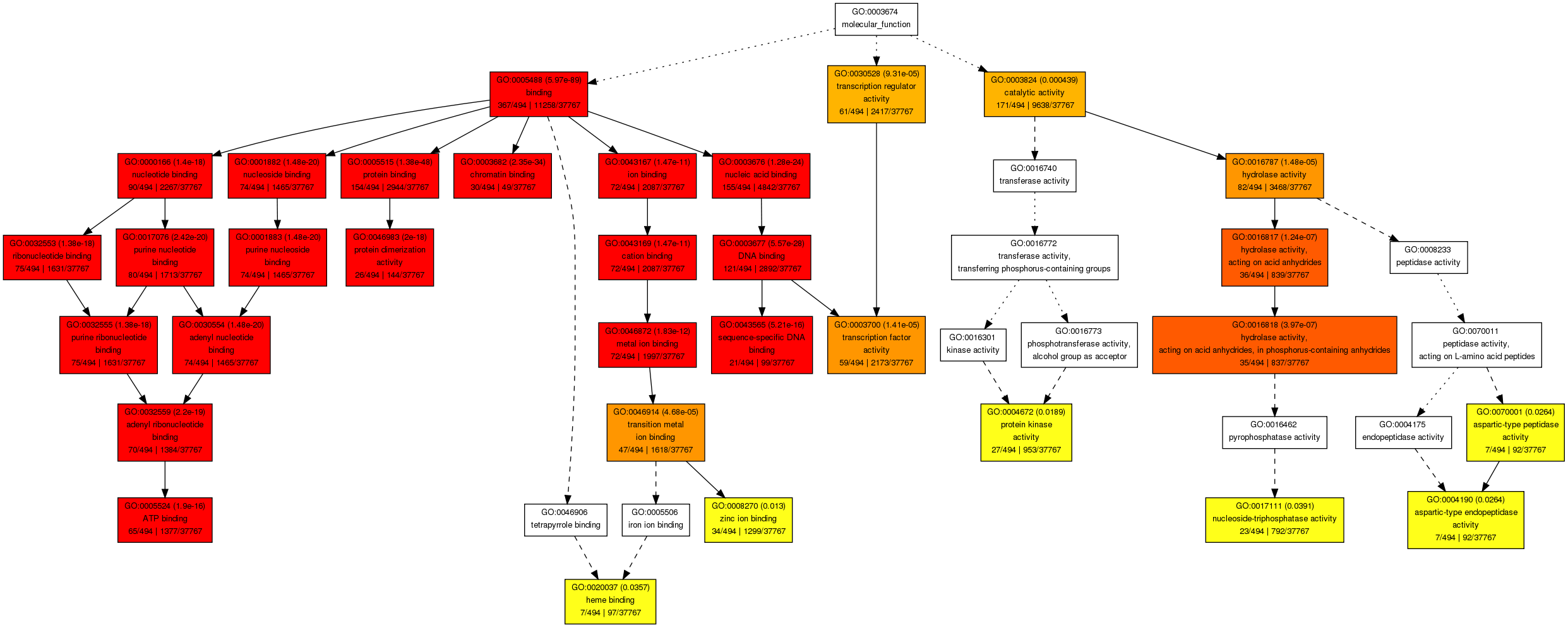

Supplement: Supplementary file 2 [file FigureS1b.PNG]

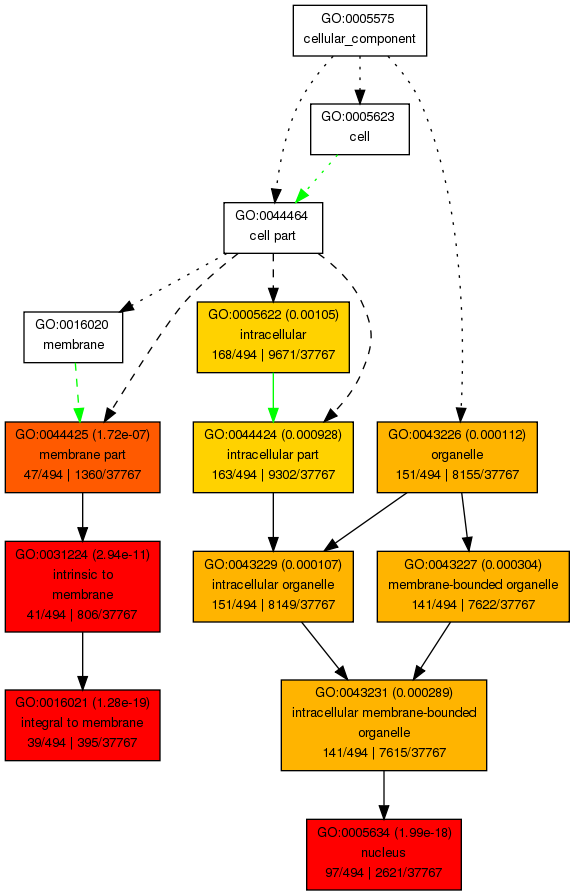

Supplement: Supplementary file 3 [file FigureS1c.PNG]

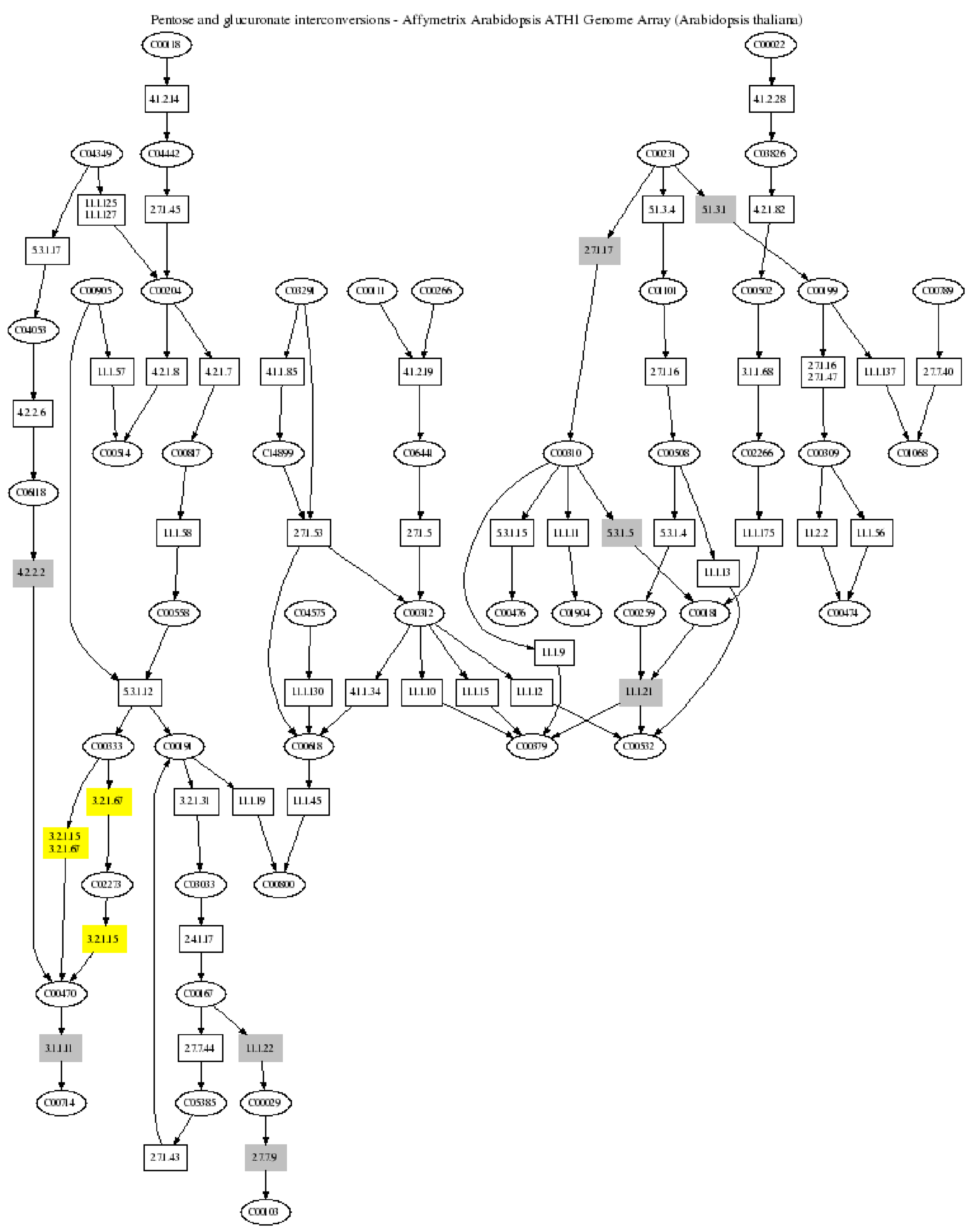

Supplement: Figure S2 — Enriched pathway (A) starch and sucrose metabolism, (B) pentose and glucuronate interconversions for target genes for predicted miRNA in B. juncea. [file FigureS2a.TIF]

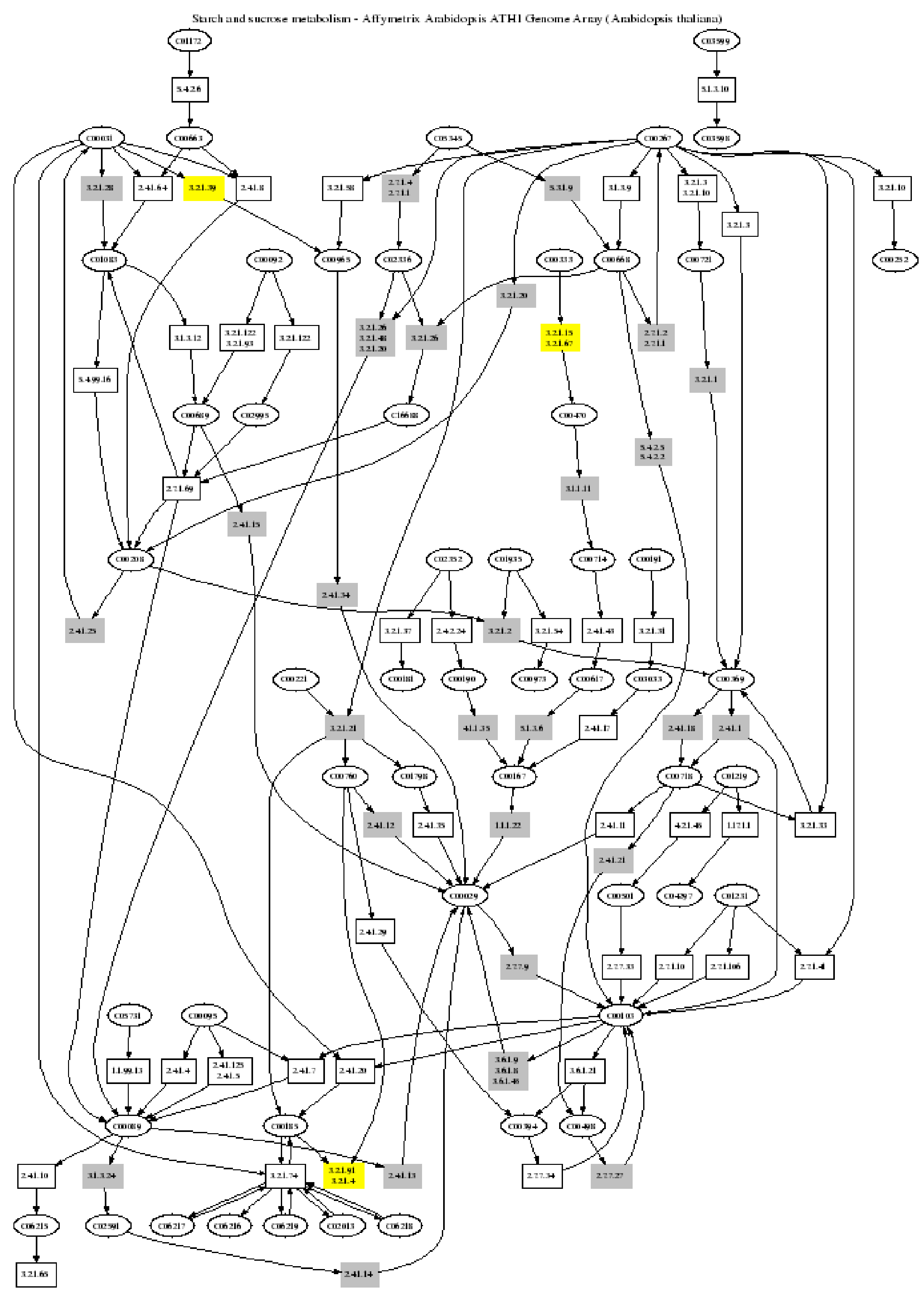

Supplement: Supplementary file 5 [file FigureS2b.TIF]

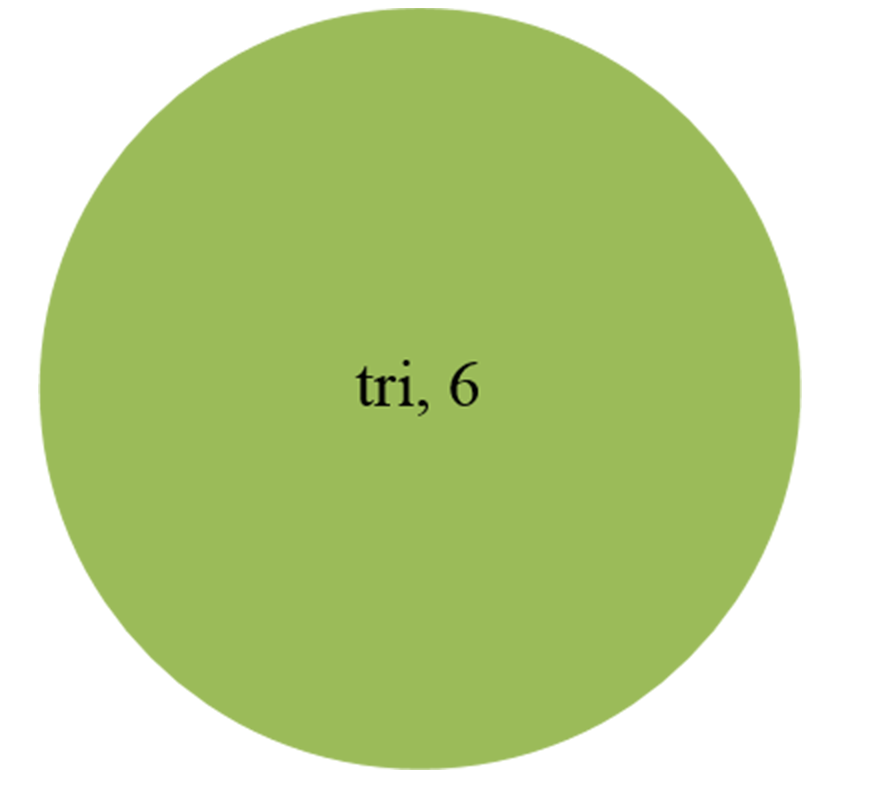

Supplement: Figure S3 — Distribution of simple sequence Repeats (SSRs) on (A) miRNA precursor sequences and (B) target genes in B. juncea. [file FigureS3a.TIF]

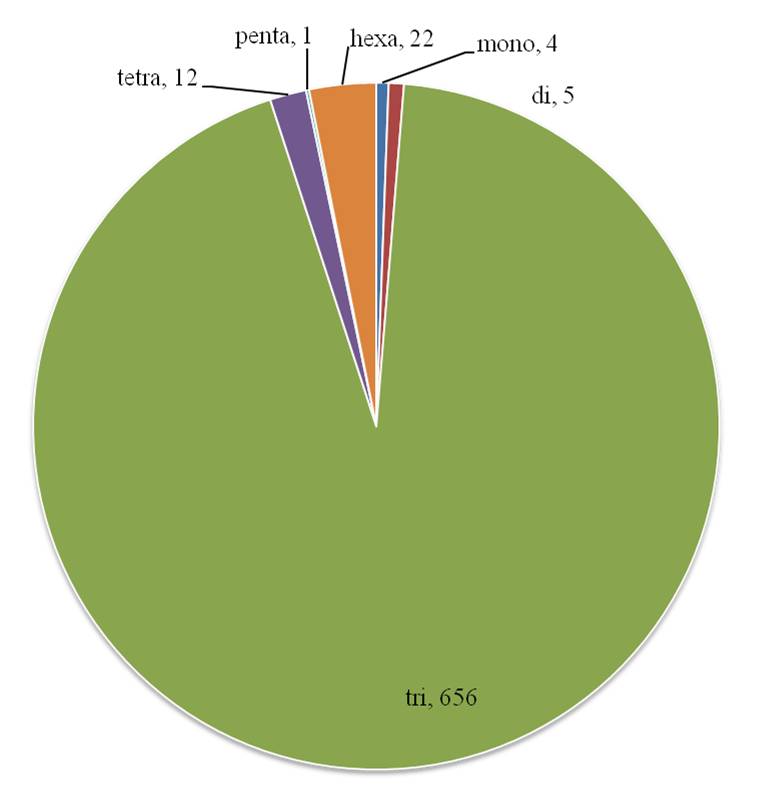

Supplement: Supplementary file 7 [file FigureS3b.JPEG]
